# Feasibility of incorporating mindfulness based mental health promotion to the pregnancy care program in Sri Lanka: a pilot study

**DOI:** 10.12688/f1000research.17049.2

**Published:** 2019-01-04

**Authors:** Thilini Agampodi, Subhashini Katumuluwa, Thulani Pattiyakumbura, Nilupulee Rankaduwa, Thushari Dissanayaka, Suneth Agampodi

**Affiliations:** 1 Department of Community Medicine, Faculty of Medicine and Allied Sciences, Rajarata University of Sri Lanka, Saliyapura, 50008, Sri Lanka

**Keywords:** Maternal mental health; Mindfulness; Pregnancy; Antenatal care; Sri Lanka

## Abstract

**Background:** Though widely discussed, mindfulness-based interventions (MBI) to improve maternal mental health is limited by lack of studies with system incorporation. We evaluate the feasibility of incorporating a MBI program into routine antenatal care (ANC) in Sri Lanka.

**Methods:** MBI included learning mindfulness concepts, practicing mindfulness sitting/reclining meditation, performing mindful movements and practicing mindfulness in daily life. Feedback from the participants were obtained through an anonymous, self-administered, semi-structured questionnaire to determine the program’s cultural appropriateness, usefulness, and feasibility.

**Results:** Participants reported that the training reduced the stress of their daily life, brought a sense of calmness to their mind and body, and improved their anger management. Participants felt strongly that this training would be very useful and a shortened version be included in the national ANC program.

**Conclusions:** This pilot study suggests that an interventional study to evaluate system incorporation of a MBI to improve maternal mental health is feasible.

## Introduction

Pregnancy and childbirth represent a time of increased vulnerability, during which a woman is exposed to many physiological and psychosocial changes. This puts pregnant and postpartum women at increased risk of mental health problems. The prevalence of mental health disorders in pregnancy and postpartum period is much greater in low-income countries (LIC) and lower-middle-income countries (LMIC) ( Fisher *et al.*, 2012) than in high income countries (HIC) ( Hendrick *et al.*, 1998). Of the mental health problems experienced by pregnant and postpartum women, antenatal depression, anxiety, and post-partum depression are the most common ( O’Hara & Swain, 1996). These mental health issues may lead to adverse pregnancy and neonatal outcomes such as small for gestational age baby ( Dejin-Karlsson *et al.*, 2000), developmental delays ( Bernard-Bonnin, 2004), poor mother-infant interaction ( Cohn & Tronick, 1989), negative affect in the infant ( Tronick & Reck, 2009), problems with cognitive development ( Singer & Fagen, 1992) and affective disorders /attention-deficit/hyperactivity disorder (ADHD) in children ( Lesesne *et al.*, 2003). This makes the antenatal period a critical time point for intervention on maternal mental health.

Despite having a strong, community-based public health care system, addressing maternal mental health has yet to gain the spotlight in Sri Lanka. The prevalence estimate for antenatal depression in Anuradhapura District, Sri Lanka is 16% ( Agampodi & Agampodi, 2013) and the national estimate for postpartum depression is as high as 27% ( Agampodi *et al.*, 2011). Furthermore, a secondary analysis of maternal death investigation reports from 2005–2011 in the North Central Province revealed that suicide was the leading cause of maternal mortality in the province, with 17.8% of maternal deaths attributed to suicide ( Agampodi *et al.*, 2014). These findings show the urgency of addressing the mental health of pregnant women in Sri Lanka.

Mindfulness is an emerging concept in mental health promotion. It was the original healing method used for centuries in ancient cultures in Asia. Clinical use of mindfulness was widely discussed in late 20 ^th^ century ( Baer, 2003; Deatherage, 1975). Several recent studies have shown that mindfulness-based interventions for pregnant women have been effective in increasing positive affect, decreasing negative affect ( Duncan & Bardacke, 2010; Vieten & Astin, 2008a), decreasing anxiety ( Duncan & Bardacke, 2010; Dunn *et al.*, 2012; Vieten & Astin, 2008a), decreasing depression ( Duncan & Bardacke, 2010; Dunn *et al.*, 2012), and decreasing stress ( Dunn *et al.*, 2012) during pregnancy. These benefits were seen to extend into the postpartum period in some cases ( Dunn *et al.*, 2012; Tomfohr-Madsen *et al.*, 2016; Vieten & Astin, 2008a). Nevertheless, two recent systematic reviews ( Dhillon *et al.*, 2017; Roy Malis *et al.*, 2017) shows mixed results on mindfulness based interventions in pregnancy. One of these reviews found that “anxiety, depression and perceived stress indicated no differences between the mindfulness intervention group and the control group” in pooled results of RCTs. However, pooled results of non-RCTs showed a “significant benefit for the mindfulness group”. Other review also showed similar results with probable effect on maternal anxiety. Both reviews suggested that more work is needed on this area of research to produce evidence-based guidelines. However, studies on integrating the mindfulness-based activities to routine program to improve maternal and newborn health outcomes are scared. We report here a feasibility study on developing and incorporating a mental health promotion program based on mindfulness for pregnant women of Sri Lanka.

## Methods

### Study location

This preliminary study was carried out in Anuradhapura, Sri Lanka. The study was planned as a part of field healthcare delivery in the field practice area of the Department of Community Medicine, Faculty of Medicine and Allied Sciences, Rajarata University of Sri Lanka. In the first phase of this study, we translated and culturally adapted the Mindful Motherhood training curriculum and in the second phase, feasibility testing was done to see whether it is possible to incorporate this training to the public health system. Both phases of the study were carried out in from May 2015 to April 2016.

### Phase I (May–July 2016)

Phase I of this study was to develop a culturally acceptable mindful motherhood training curriculum. We used the “Mindful Motherhood” training curriculum ( Vieten, 2009), published by the Institute of Noetic Sciences (IONS) and made available for professional use for our training programme. The general structure and content of the IONS training was left the same; however, some of the reading materials and activities were modified to be more relevant and acceptable to a Sri Lankan population. In addition, supplemental readings were added from other sources. In the Sri Lankan community, “loving kind medication” is a well-established practice. We included supplementary reading and practice component of loving kind medication in to all eight sessions. The sources used were simple Buddhist teaching, without addition religious components. The changes were done after extensive discussions among three community physicians, a public health physician, maternal health service providers at divisional and grassroots level and medical officers. All supplemental readings were translated from English to Sinhalese, the main language spoken in Sri Lanka. Translations were performed by two medical graduates, who were fluent in both languages as well as mindfulness teaching. Tamil translations were not made at this time, as the majority population of the Nuweragam Palath Central (NPC) region, where the study was conducted, speaks Sinhalese. Back translation was not done, as the procedure was rather a cultural adaptation than tool translation. However, three investigators individually looked at the translations and discussed and finalized the materials.

The aim of our programme was to introduce the concept of mindfulness and develop the skills of the pregnant mothers to be mindful in day-to-day life, with the ultimate goal of promoting mental well-being. During the programme development, four authors (T.A., S.K., T.P. and N.R.) who did the training, had undergone mindful training. Three of the authors involved in the training (T.A., S.K. and N.R.) were regular practitioners of Buddhist mindfulness in daily life. Our curriculum consisted of eight, 2- to 3-hour weekly training sessions. Each session consisted of several components; introduction of a short mindfulness concept; practice of mindfulness sitting/reclining meditation (i.e. breathing meditation, meditation with focus on thoughts/feelings/sensations, and several types of body scans); performing mindful movement (i.e. prenatal yoga, mindful walking, simple mindful movement series); and sometimes a mindfulness in daily life activity or discussion (i.e. mindful eating, how to complete daily tasks mindfully, mindfulness in relationships). In addition to the weekly sessions, each week the women were provided with readings summarizing or related to the week’s mindfulness concepts. The initial 8-week curriculum, supplementary materials, and all training related documents are publically available on OSF ( Agampodi *et al.*, 2018a).

### Phase II (August 2015–April 2016)

In the second phase, we field-tested the feasibility of incorporating a mindfulness-training program into routine antenatal care (ANC) in Sri Lanka. For the intervention, we recruited pregnant women at less than 32 weeks gestational age, and who were able to read and write Sinhalese, from the NPC region. Those who were not permanent residents and planning to leave the area for delivery (in Sri Lanka, some females go to their parents’ place for delivery) were excluded from the study. Participants were recruited at the antennal clinics. We also included the Public Health Midwife (PHM), the grassroots level healthcare provider for pregnant women in the program. The PHM was included because she has extensive experience working with pregnant women in rural areas and has a unique understanding of their needs and perspectives on a range of pregnancy-related issues, providing valuable information regarding the feasibility of our training. In addition, if any intervention to be included into the routine public health system in Sri Lanka, it needs to be carried out by the PHM. The PHM selected the participants for the study purposefully, based on interest and availability to participate. Pregnant women with a history of mental disorder with a psychotic component were excluded from the study. The sample size was predetermined (not calculated) as 12–15 participants, to ensure an adequate level of interaction between the participants and instructors and based on feasibility to visit the mindfulness-training center at Faculty of Medicine and Allied Sciences.

In addition to attending the weekly training sessions developed in phase 1, as a homework assignment, women were asked to practice each of the mindfulness practices learned in the session at home. The women recorded their practice on a weekly record sheet and handed it in the following week. They were also asked to keep a diary and write any thoughts or feelings about the sessions and how they felt during the week while practicing. After seven weeks of the program, participants were given an anonymous, detailed, self-administered, semi-structured questionnaire to obtain their feedback. The questionnaire sought to gain the participants’ detailed insights into how the program affected their lives (if at all), the most useful aspects of the training, their suggestions for improvement, and whether and how a similar training should be incorporated into the current ANC program. We ask about acceptability of the programme and also feasibility of practicing it at home. No maternal mental health outcome variables were evaluated in this pilot, as it was only a feasibility study.

To evaluate the feasibility and to triangulate data for future system incorporation, facilitators of the training also recorded their thoughts on the program content, structure, and the logistics of conducting. The self-reported descriptions were collected separately to analyses and thematic analysis was done for these feedbacks as well.

### Ethical approval

Ethical clearance was obtained from the Ethics Review Committee, Faculty of Medicine and Allied Sciences, Rajarata University of Sri Lanka (ERC/2015/15). Written informed consent was obtained from all the participants included in the study.

## Results

In total, 12 women were recruited and participated in the training. None of them had known psychiatric problems. The women ranged in age from 18 to 30 years, and were between 10 and 27 weeks gestational age. After completion of the program, participants described many benefits they received through the training. A total of eight participants completed the diaries and the feedback.

## Perceived psychological benefits

### Daily life stress

Most of the women (7/8) reported that the training reduced daily life stressors and brought a sense of calmness to their mind. All the women stated that participating in the training had also improved the way they responded to stressful situations.

*“I was bored and stressed with the daily routine. With the training I was able to face day-to-day problems in a calm mindful manner.”* 22-year-old pregnant female.

### Improvement in personal communication

The women reported improvements in interpersonal communication. The training had helped them to improve the way they interacted with their spouse and/or current child(ren), or that their view of how best to interact with their yet unborn child had changed based on what they had learned.

*“When my elder child was stubborn, I was able to modestly tackle it. Even when other people came to argue, I was able to face them with patience. It was successful.”* 30-year-old pregnant female.

### Controlling emotions

Some women also reported that the training helped them to control their emotions, with several (3 of 8) stating that they were better able to control their anger.

*“My elder child had a fall and was injured. All the family members were panicked. I was able to remain calm and do the needful. I was not like this before. This was a new experience for me. I acquired this skill from the training.”* 28-year-old pregnant female.

### Improving quality of work and life

The women told that they were able to do their duties at home and work place in a more efficient manner than in their past.

*“Now I’m able to do each and every task with more understanding. This gives me clarity and calmness in mind. I feel less exhausted. I’m able to sleep well.”* 18-year-old pregnant female.

## Perceived physical benefits

In addition to the mental and emotional benefits, all the women reported physical benefits, including feeling a sense of comfort and relaxation for the body when practicing the mindful movement at home. One of the women reported successfully using several of the meditation and body scan techniques help her cope with an experience of abdominal pain, stating,

*“when I fell ill and I started practicing the things we learned, putting them into action one by one, the body which was full of pain became normal in an unbelievable way.”* 25-year-old pregnant female.

Every participant reported that they intended to continue the daily practice of various techniques to cultivate mindfulness, learned during the training.

## Evaluation of content

Participants evaluated the different components or strategies used to cultivate mindfulness during this training. The mindful movement series was the most popular strategy among women. Of the mindfulness concepts taught, the women found the topics of “introduction to mindfulness,” “staying in the present moment,” “acceptance,” and “avoiding controlling and judging” to be the most interesting and helpful in their daily lives. Of the sitting/lying down meditations, women enjoyed and perceived the most usefulness from the body scan, loving kindness meditation, and mindful breathing practice. Everyone reported that they found the “Mindful Mother Check-in” cards (cards given to them at the beginning of the training with simple steps to bring the mind to the present moment applying the practices they had learned), which they posted around their home, to be helpful to calm their mind, particularly during stressful situations. Of the mindful movements taught, the majority of the women (6 of 8) enjoyed the short mindful movement series the most.

In addition to participant feedback, the facilitators of the training also recorded their thoughts on the program content, structure, and the logistics of conducting it. We experienced that the depth of the material being allocated for each session was advance, so that it cannot be grasped with just a simple explanation for a participant who missed a session. For this same reason, we found it a challenge to complete all the material in the allotted 2-hour time, often running over. Additionally, the women seldom asked questions about the material (which may be a cultural factor) or for clarification during the sessions.

## Feasibility and timing

Each participant felt time allocated for each of the training segments was appropriate, except for one who felt it would be better if more time was given for the mindful movement section. Although we took into consideration the demands on the average pregnant woman in our area when deciding a meeting time, we still found that it was very difficult to get the women to come consistently to every session. This is likely due to several cultural factors. In Sri Lanka, many women in more rural areas are stay-at-home wives/mothers, taking care of the household and the children. So unless there is someone at home, usually a relative, who can watch the children or pick them up from school while the mother is out, she may not be able to come, may come late or need to leave early, which we experienced on multiple occasions. All of the women also had to take public transportation to get to the trainings, which added to the challenge.

## Feasibility of incorporation into ANC system

Women were asked their thoughts on the potential benefit of incorporating a mindfulness training into routine ANC in Sri Lanka. All the participants felt that this training would be very useful and important for other pregnant women. While agreeing with this, one woman raised the concern of whether one two-hour session every three months would provide sufficient time and guidance for women to fully understand the meaning and feel the value of the concepts taught. It was also suggested that it would be beneficial to have the husbands of the pregnant women involved in the program as well. Overall, all the participants, including the PHM, felt that it would be beneficial and feasible to incorporate a mindfulness program into the national ANC system if the current curriculum was shortened and included in the once per trimester antenatal sessions that are already part of the ANC system in Sri Lanka.

Based on Phase I and Phase II of this feasibility assessment, we prepared the final version of intervention. The final structure of the mindfulness based intervention to improve mental health of pregnant mothers is illustrated in Table 1.

**Table 1.** Proposed 8-weeks mindfulness-based training program to be tested in a larger study in Sri Lanka for pregnant women.

Session	Theme	Activities	Duration
Day 1	Introduction to mindful motherhood training	Introduction to mindfulness and mindful motherhood training	30 minutes
		Mindful daily awareness using mindful eating of a simple food	15 minutes
		Introduce mindful movement	15 minutes
		Introduce mindful sitting meditation and awareness in breathing	15 minutes
		Loving kindness meditation	15 minutes
Day 2	Control vs mindfulness, what is mindfulness? Acceptance/Willingness	Mindful movements	20 minutes
		Recap the purpose of mindful awareness Explain further mindfulness	20 minutes
		Concept: acceptance	15 minutes
		Mindful awareness of breathing	15 minutes
		Introduce mindful mothers’ check-in	10 minutes
		Loving kindness meditation	15 minutes
Day 3	The observing self	Mindful movement practice	20 minutes
		Mindful mothers’ check-in	10 minutes
		Concept: observing self	30 minutes
		Mindful awareness practice (sitting) -body scan	20 minutes
		Loving kindness meditation	15 minutes
Day 4	Train of thoughts Metacognition	Mindful walking exercise	20 minutes
		Mindful mothers’ check-in	10 minutes
		Concept: Train of thoughts	30 minutes
		Observing the activities of mind	10 minutes
		Observing thoughts	20 minutes
		Loving kindness meditation	15 minutes
Day 5	Present movement focus	Mindful movement and body scan	30 minutes
		Mindful mothers’ check-in	10 minutes
		Concept: present movement focus	30 minutes
		Mindful awareness of present movement sitting meditation	20 minutes
		Loving kindness meditation	15 minutes
Day 6	Additional qualities of mindfulness	Exercise to identify mindful/unmindful moments	20 minutes
		Concept: qualities of mindfulness (3 new qualities) nonstriving, beginners mind, being vs. doing	30 minutes
		Mindful movement – walking around the place	20 minutes
		Mindful awareness of thoughts, feelings and body sensation	20 minutes
		Loving kindness meditation	15 minutes
Day 7	Self-compassion	Mindful movement and body scan	20 minutes
		Mindful mothers’ check-in	10 minutes
		Concept: Self-compassion	30 minutes
		Self-compassion – practical exercises and discussion	30 minutes
		Refresh concepts learnt so far	15 minutes
		Loving kindness meditation	15 minutes
Day 8	Mindful awareness in everyday life Mindfulness in childbirth	Mindful movement	15 minutes
		Concept: wise action and intention	30 minutes
		Quick awareness and action exercise	15 minutes
		Mindful decision making	20 minutes
		Mindful awareness as a refuse and source of strength	15 minutes
		Mindful mom’s promise	10 minutes
		Mindful motherhood as a whole	15 minutes

## Discussion

This preliminary study shows that a incorporating a mindfulness based mental health program for pregnant mothers to the pregnancy care program in Sri Lanka is feasible and culturally acceptable. It also suggests that learning mindfulness concepts and the techniques to cultivate mindfulness may improve a woman’s ability to cope with stress during her pregnancy. Important future steps should include efforts to capture the latter outcome quantitatively, as has been done in prior pilot studies ( Duncan & Bardacke, 2010; Dunn *et al.*, 2012; Vieten & Astin, 2008a).

The program developed in this pilot study was based on the standard 8-week mindfulness intervention used in many other previous studies ( Astin *et al.*, 2003)( Byrne *et al.*, 2014)( Dunn *et al.*, 2012)( Vieten & Astin, 2008b). However, a brief mindfulness interventions even for a 3-week duration has proven the potential usefulness of mindfulness interventions ( Beattie *et al.*, 2017; Matvienko-Sikar & Dockray, 2017). Although there are number of studies on mindfulness interventions, there is not enough evidence found about the most effective duration for a mindfulness program. Thus, further studies has to be conducted in the future to assess the effective time duration for mindfulness interventions. Taking into account the challenges the facilitators experienced during the intervention, trying to cover all the material in a comprehensible manner within the allotted 16-hour time period of the training may not be feasible. Careful consideration will be needed to select some of these concepts that could practically be covered during the three ANC sessions, and would likely be of greatest benefit and/or interest to the pregnant women. One has to bear in mind that the system integration requires assessment of operational and other aspects; not only the field health worker feedback. However, the feedback from our pilot study participants will be valuable when making these decisions. Though we have not assessed mental health outcomes after the intervention, which was beyond the scope of this study, the reported perceived psychological benefits were positive and noteworthy.

Given the positive response from our small feasibility pilot, the next steps will include using the participants’ feedback to create a modified, shortened mindfulness training curriculum for pregnant women, and running a larger field study using this revised curriculum. Maternal mental health outcomes should also be evaluated using pre- and post-intervention questionnaires that have been validated for use in Sri Lankan populations. This will be an important step in establishing any effect that the mindfulness training may have on maternal mental health-related outcomes, including distress, depression, and coping skills. The possibility of any benefits of the training extending into the postpartum period, as suggested by pilot studies ( Roy Malis *et al.*, 2017; Vieten & Astin, 2008a) should also be evaluated. The degree of success of launching such a program nationally, or even locally, will depend heavily on the willingness and enthusiasm of the PHMs, as eventually, if the program is to be sustainable, it will be trained PHMs who will be leading each of the mindfulness sessions. To date, the support from the local PHM community and Medical Officer of Health (MOH) has been strong for this program. When presented an overview of the program and the evidence behind the teachings, many of the PHMs were visibly enthusiastic about the benefits such a program could have for their patients and were eager to offer their support.

The main limitation of our study was the small sample size. Proper feasibility assessment is difficult with a sample of 12 pregnant women. There are many variables to assess in relation to feasibility. In addition, the inconsistent attendance of some of the participants, and having a sample of only Sinhalese-speaking women also limits the generalizability of the pilot work. To serve both the Sinhalese and Tamil populations in Sri Lanka, the curriculum will also need to be translated and tested in a Tamil-speaking population.

## Conclusion

This pilot study suggests that a mindfulness-training program is a culturally appropriate It also shows that it is feasible to do a proper trial before deciding on up scaling and system incorporation. Further research must investigate the effectiveness of a shortened, modified program on impacting maternal mental health-related outcomes during pregnancy and beyond.

## Data availability

### Underlying data

Raw data from the participants’ answers to the questionnaires are available on OSF. Please note that data are provided in Sinhalese. DOI: https://doi.org/10.17605/OSF.IO/QFA6X ( Agampodi *et al.*, 2018b).

### Extended data

All documents related to the project, including the initial proposal, ERC certificate, questionnaire (with translation) and training material are available on OSF. DOI: https://doi.org/10.17605/OSF.IO/45HNR ( Agampodi *et al.*, 2018a).

All data are available under the terms of the Creative Commons Zero “No rights reserved” data waiver (CC0 1.0 Public domain dedication).
