# Supplementary material for: Feasibility of incorporating mindfulness based mental health promotion to the pregnancy care program in Sri Lanka: a pilot study
Source: F1000Res. 2019 Jan 4;7:1850. Originally published 2018 Nov 27. [Version 2] doi: 10.12688/f1000research.17049.2 (PMC7096187; doi:10.12688/f1000research.17049.2)
Supplement: Peer review history

# Peer review: Reviewer response for version 2

**DOI:** 10.5256/f1000research.19388.r57328

**Published:** 2019-12-17

**Authors:** Alys Einion-Waller

**Affiliations:** 1 Department of Interprofessional Health Studies, Swansea University, Swansea, UK

The article summarises a feasibility study to include mindfulness activities to support maternal mental health in pregnancy, incorporating this into a pregnancy care programme in Sri Lanka. Please ensure clearer signposting of the literature reviewed (e.g. paragraph 1, column 2, page 3).

 More details of how the tool used was 'culturally adapted' would aid in replicability or would provide more information for healthcare providers to transfer this intervention to other cultural settings. The positive results suggest that continuing to test this intervention would be appropriate, but given the small sample size, caution should be used in transferring these findings to other settings.

I confirm that I have read this submission and believe that I have an appropriate level of expertise to confirm that it is of an acceptable scientific standard.

# Peer review: Reviewer response for version 2

**DOI:** 10.5256/f1000research.19388.r42551

**Published:** 2019-02-08

**Authors:** Moraendage Wasantha Gunathunga

**Affiliations:** 1 Department of Community Medicine, Faculty of Medicine, University of Colombo, Colombo, Sri Lanka

I read the revised article. As suggested revisions have been made I have no further comments or revisions to be suggested.

I confirm that I have read this submission and believe that I have an appropriate level of expertise to confirm that it is of an acceptable scientific standard.

# Peer review: Reviewer response for version 1

**DOI:** 10.5256/f1000research.18639.r41129

**Published:** 2018-12-17

**Authors:** Moraendage Wasantha Gunathunga, Aruni Gallage

**Affiliations:** 1 Department of Community Medicine, Faculty of Medicine, University of Colombo, Colombo, Sri Lanka

Maternal health is an important concern in Sri Lanka as in many other countries. Though maternal well being is addressed with vigor in Sri Lanka mental well being is not equally addressed. Hence, exploring possibilities of incorporating mindfulness into maternal care services is of much importance and has the potential to contribute significantly to scientific literature. It is commendable that authors embarked on this much needed research.

 In phase one, what the authors mean by “Buddhist teaching, without addition religious components” is unclear. If any use can be obtained, for health benefits  from any religious material we do not see any reason of not using it and acknowledging it.

 Among those who took part in extensive discussions no mention is made about experience in actually practicing mindfulness. The fact that three researchers have been trained in mindfulness and are regular practitioners is noteworthy. This is essential as mindfulness involves a phenomenological component. Without substantial direct experience researchers will not understand certain components of behaviours related to mindfulness. It is also required to mention whether the researchers are capable of dealing with advanced practitioners’ queries or such services are made available when needed. This is important as all mothers who start mindfulness practice are not at the same baseline and progress is not uniform. They can be expected to behave very close to a normal distribution where some progress very fast and needing advanced help.

 In phase two “Pregnant women with a history of mental disorder with a psychotic component were excluded from the study”. It is unclear why only psychotic component was excluded. It would have been more appropriate to exclude those who have a known psychiatric illness in this phase.

 It has been mentioned that mental health variables were not assessed. However, perceived psychological benefits were assessed and positive. It is too early to count on these as the benefits cannot be directly attributed to the mindfulness practice in this design. This association, though, is noteworthy.

 The sample size of 12 is inadequate to have a sufficient idea on the feasibility of the intervention due to the wide variation in the variable related to feasibility.

  As mentioned in “Feasibility of incorporating into Antenatal Care(ANC) system” Participants opinion regarding incorporating this intervention to national antenatal care in Sri Lanka has to be taken with reservation as they do not see the operational aspects of such a program when it is scaled up. In the responses there is also an element of social desirability bias.

 “The degree of success of launching such a program nationally, or even locally, will depend heavily on the willingness and enthusiasm of the Public Health Midwive(PHM)s, as eventually, if the program is to be sustainable, it will be trained PHMs who will be leading each of the mindfulness session”. This is asking too much from already over burdened PHM in Sri Lanka. Our experience is that, out of the numbers who take up mindfulness training, only a small proportion will qualify to be trainers. Hence we consider planning to train PHMs as trainers is a weakness in the design.

 As mentioned in the discussion inconsistent attendance can be a difficult challenge to meet as take-up of this kind of interventions by different individuals vary widely.

 It is still premature to conclude “..a feasible way to incorporate promotion of maternal mental health into routine ANC in Sri Lanka”. Though it appears feasible to implement the intervention whether it improves mental well being as an outcome has not been assessed in this work. Hence this claim is not justified.

 In the abstract, there is no conclusion in relation to the set objective of evaluating feasibility of intervention.

 Overall, considering the dearth of literature in this area of research and the importance in relation to promotion of maternal mental well being this work warrants indexing with modifications.

We confirm that we have read this submission and believe that we have an appropriate level of expertise to confirm that it is of an acceptable scientific standard, however we have significant reservations, as outlined above.

# Response

**Published:** 2018-12-29

**Authors:** Suneth Agampodi

**Affiliations:** Health, PGIM, Sri Lanka

**Reply to reviewer’s comments**

We would like to appreciate the comments by the reviewer and it definitely helped us to enhance the quality of this manuscript. Based on the points raised by the reviewer, we have revised the manuscript.  The point by point explanations for reviewers comments are below.

**In phase one, what the authors mean by “Buddhist teaching, without addition religious components” is unclear. If any use can be obtained, for health benefits from any religious material we do not see any reason of not using it and acknowledging it.**

In the phase one we had several meetings with stakeholders, before starting the project. Service providers had a concern that this intervention looks as a Buddhist intervention. To overcome this barrier, we mentioned the term “mindfulness” though the teaching and training were obtained from Buddhist practices. We acknowledge the materials in the article, but not during the training. 

**Among those who took part in extensive discussions no mention is made about experience in actually practicing mindfulness. The fact that three researchers have been trained in mindfulness and are regular practitioners is noteworthy. This is essential as mindfulness involves a phenomenological component. Without substantial direct experience researchers will not understand certain components of behaviours related to mindfulness. It is also required to mention whether the researchers are capable of dealing with advanced practitioners’ queries or such services are made available when needed. This is important as all mothers who start mindfulness practice are not at the same baseline and progress is not uniform. They can be expected to behave very close to a normal distribution where some progress very fast and needing advanced help.**

Of the six authors, three were practicing mindfulness for a long period. However, it is difficult to reply to this comment about needing advanced help. The purpose of this training was to improve day to day mindful practices and to help the pregnant mothers to practice it up to a certain extent. It was neither our intention to go up to the advance mindful practices nor in assisting pregnant mothers who are already having such practices, because it was beyond any routine intervention. 

**In phase two “Pregnant women with a history of mental disorder with a psychotic component were excluded from the study”. It is unclear why only psychotic component was excluded. It would have been more appropriate to exclude those who have a known psychiatric illness in this phase.**

In the research protocol, we had this as an exclusion criteria, but none of the selected pregnant mothers had psychiatric illnesses. We revised the manuscript to explain this. 

**It has been mentioned that mental health variables were not assessed. However, perceived psychological benefits were assessed and positive. It is too early to count on these as the benefits cannot be directly attributed to the mindfulness practice in this design. This association, though, is noteworthy.**

We fully agree with this comment. We got a qualitative feedback and a proper trial is required to comment on the benefits. We have included this suggestion in the discussion.

**The sample size of 12 is inadequate to have a sufficient idea on the feasibility of the intervention due to the wide variation in the variable related to feasibility.**

This also we agree fully. This was a “pilot study’ to assess feasibility as mentioned in the title. The study is not even a full feasibility study. We emphasize this in the revised limitation component of the manuscript. 

**As mentioned in “Feasibility of incorporating into Antenatal Care (ANC) system” Participants opinion regarding incorporating this intervention to national antenatal care in Sri Lanka has to be taken with reservation as they do not see the operational aspects of such a program when it is scaled up. In the responses there is also an element of social desirability bias.**

Again we agree with this comment fully and included in the discussion. 

**“The degree of success of launching such a program nationally, or even locally, will depend heavily on the willingness and enthusiasm of the Public Health Midwive (PHM)s, as eventually, if the program is to be sustainable, it will be trained PHMs who will be leading each of the mindfulness session”. This is asking too much from already over burdened PHM in Sri Lanka. Our experience is that, out of the numbers who take up mindfulness training, only a small proportion will qualify to be trainers. Hence, we consider planning to train PHMs as trainers is a weakness in the design.**

First, we also share the same concern about overburden PHM duties. At the same time, in Sri Lanka, national maternal health intervention through other means has never had sustainability. The second point is well taken and only a small proportion will be qualify to  be mindfulness trainers; not only among PHMs but also among other groups. This limitation and challenge was added to the revised discussion. 

**As mentioned in the discussion inconsistent attendance can be a difficult challenge to meet as take-up of this kind of interventions by different individuals vary widely.**

Agree and it's already in the discussion. 

**It is still premature to conclude “..a feasible way to incorporate promotion of maternal mental health into routine ANC in Sri Lanka”. Though it appears feasible to implement the intervention whether it improves mental well being as an outcome has not been assessed in this work. Hence this claim is not justified.**

Agree and this conclusion was revised.

**In the abstract, there is no conclusion in relation to the set objective of evaluating feasibility of intervention.**

Conclusion of the abstract is revised.
